# Thrombocytosis in the Setting of Isomerism and a Functionally Univentricular Heart

**DOI:** 10.7759/cureus.383

**Published:** 2015-11-19

**Authors:** Rohit Loomba

**Affiliations:** 1 Department of Cardiology, Children's Hospital of Wisconsin

**Keywords:** thrombocytosis, heterotaxy, isomerism, single ventricle, univentricular, platelet

## Abstract

Introduction

Bodily isomerism, also known as heterotaxy, is a unique entity in which there is mirror imagery in various organ systems. Those with isomerism will often have congenital malformations of the heart requiring functionally univentricular palliation. Anecdotally, thrombocytosis has been noted with higher frequency in patients with isomerism. This study aimed to determine the prevalence of thrombocytosis at different stages and identify independent predictors of thrombocytosis.

Methods

We identified patients with isomerism and a functionally univentricular heart who received care at our institution between January 1998 and January 2014. Clinical data regarding these patients was collected via chart review. Platelet counts were collected before initial surgical palliation, the day prior to second surgical palliation, and the day prior to the third surgical palliation. Platelet counts from the first postoperative day following all three surgical palliations was also collected. Mean platelet counts were compared between consecutive stages as well as to the initial platelet count. The frequency of thrombocytosis was also calculated at each point with a binomial logistic regression conducted to determine independent risk factors of thrombocytosis at each time point.

Results

A total of 57 patients were included in the analysis. The mean platelet count before initial surgical palliation was 349.21 x 10^9^/L and decreased with age. Thrombocytosis was noted in 15.8% prior to initial surgical palliation and 23.6% prior to second surgical palliation. Thrombocytosis was no longer noted after second surgical palliation. No independent risk factors for thrombocytosis were identified.

Conclusion

Thrombocytosis is not infrequent during the first year of life in those with isomerism. It is important to be vigilant of platelet counts in this population as thrombocytosis may lead to increased thromboembolic events, particularly in the setting of a Blalock-Taussig shunt. Thrombocytosis nearly always resolves after the second surgical palliation.

## Introduction

Bodily isomerism, also referred to as heterotaxy, is a multisystem laterality defect affecting nearly 1 in 10,000 children [1-3]. In the presence of isomerism, there is mirror imagery of the thoracic organs such that there are morphologically left or right bronchi bilaterally as well as morphologically left or right lungs bilaterally. Also noted in the thoracic cavity is isomerism of the atrial appendages. Abnormalities in other organ systems are also present with abnormal lateralization but not isomerism present in the abdominal cavity. Segregated on the basis of atrial appendage morphology into right or left isomerism, those with bodily isomerism (hereafter referred to as simply isomerism) will have a different constellation of findings based on the sidedness of isomerism [4-7]. Congenital malformations of the heart are common with many requiring functionally univentricular palliation. Abnormalities in the cardiac conduction system are also common with subsequent development of arrhythmias [8-10]. Those with isomerism will often have splenic abnormalities, the common presence of multiple spleens in left isomerism and absence of a spleen in right isomerism. Some, however, may have a solitary, normally located spleen. Regardless of splenic anatomy, however, functional asplenia may be present [11]. Although the precise mechanism of this has not been elucidated, it is possible that ciliary dyskinesia may play a role in this.

As a consequence of functional asplenia, those with isomerism are at increased risk for bacteremia, particularly in the first five years of life. Children with isomerism and functionally univentricular hearts have also been demonstrated to have higher platelet counts, which may also be secondary to functional asplenia. This appears to be associated with an increased risk of thrombosis, particularly in patients with Blalock-Taussig shunts (subclavian artery to pulmonary artery shunt) [12]. We reviewed our institutional experience with patients with isomerism and functionally univentricular hearts to demonstrate platelet count at various stages of life and identify factors associated with thrombocytosis.

## Materials and methods

We identified patients with isomerism who were cared for at our institution from January 1998 to January 2014. Data regarding their cardiac and noncardiac anatomy, cardiac surgical palliations, and other findings associated with isomerism were collected by chart review. No patients who had a functionally univentricular heart and isomerism were excluded. Approval for this study was obtained from the Children's Hospital of Wisconsin Institutional Review Board. As this was a retrospective review, a consent waiver was granted for this study.

Cardiac anatomy was analyzed by use of echocardiography although findings were confirmed with catheterization studies, computed tomography, and magnetic resonance imaging when available. This principle of sequential segmental analysis was used to analyze the cardiac anatomy [13]. Bronchial morphology was determined using chest radiographs, computed tomography, and magnetic resonance imaging [14-15]. Intestinal malrotation was assessed by the review of upper gastrointestinal studies. Splenic status was analyzed most often by abdominal ultrasonography, but some patients also had computed tomography or magnetic resonance imaging from which splenic anatomy could also be noted. As it wasn’t routine during our study period for the surgeons to document what the atrial appendage morphology was noted to be at the time of surgery and the difficulty associated with imaging the appendages, this data was not available. Instead, we used an aggregate of all the collected features to segregate the entire cohort into having right or left atrial appendage morphology.

Data regarding surgical palliation was obtained from the operative notes. This included the precise procedure, cardiopulmonary bypass time, aortic cross-clamp time, and any operative complications. Whether or not patients were extubated in the operating room or not was also noted. The total postoperative length of mechanical ventilation required as well as the need for reintubation in the postoperative period was also collected. The total length of stay associated with surgical palliation was also collected.

Platelet counts were obtained from complete blood counts that are routinely obtained on the day prior to surgery as part of the preoperative assessment. Complete blood counts are also routinely obtained postoperatively and were used for analysis.

The mean platelet count was calculated between birth and first surgical palliation, the day after initial surgical palliation, the day prior to second surgical palliation, the day after second surgical palliation, the day prior to third surgical palliation, and the day after third surgical palliation. Mean platelet count was also calculated at least one year after the third surgical palliation. A student t-test was used to compare mean platelet count between consecutive time points (i.e., before initial surgical palliation and postoperative day 1 after initial surgical palliation). A student t-test was also used to compare mean platelet count at every point to the platelet count before initial surgical palliation.

Thrombocytosis was defined as having a platelet count of 450 x 10^9^/L. The percent of those having thrombocytosis at each time point was also calculated and compared. A binomial logistic regression was conducted at each time point to determine risk factors for thrombocytosis.

Statistical analysis was done using SPSS version 20.0 (Chicago, IL). A p-value of less than 0.05 was considered statistically significant.

## Results

A total of 57 patients with isomerism and functionally univentricular hearts were included in the analysis. Of these, 54.5% were male. Cardiac findings included common atrioventricular junction in 68.4% and double outlet right ventricle in 47.4%. Discordant ventriculoarterial connections were present in 24.6%. Right isomerism was present in 70.2% and left isomerism in 29.8%. An absence of the spleen was noted in 73.7%, presence of multiple spleens in 14.0%, and the presence of a solitary, normally located spleen in 10.5% (Table 1).

**Table 1:** Overall characteristcs Patient characteristics for those included in the analysis

Characteristic	Frequency (Percent) or Median (Minimum to Maximum)
Total number	57
Male	31 (54.5)
Common atrioventricular junction	39 (68.4)
Double outlet right ventricle	27 (47.4)
Double inlet left ventricle	1 (1.8)
Left sided superior caval vein	34 (59.6)
Bilateral superior caval veins	29 (50.9)
Interrupted inferior caval vein	12 (21.1)
Discordant ventriculoarterial connections	14 (24.6)
Splenic anatomy	
Absence of a spleen	42 (73.7)
Multiple spleens	8 (14.0)
Solitary spleen	6 (10.5)
Data not available	1 (1.8)
Evaluation for intestinal malrotation	38 (66.7)
Intestinal malrotation	24 (43.9)
Surgery for intestinal malrotation	23 (40.4)
Morphology of the atrial appendages by inference	
Left	17 (29.8)
Right	40 (70.2)
Bronchial morphology	
Left	5 (8.8)
Right	45 (78.9)
Imaging not available to determine	7 (12.3)
Abdominal situs	
Right sided liver, left-sided stomach	19 (33.3)
Left sided liver, right-sided stomach	13 (22.8)
Midline liver with right or left-sided stomach	13 (22.8)
Data not available	4 (7.0)
Arrhythmia	24 (42.1)
Need for pacemaker	6 (10.5)
Need for extracorporeal membrane oxygenation	7 (12.3)
Episode of documented bacteremia	11 (19.3)
Prenatal diagnosis	39 (68.4)
Age at initial surgical palliation (n=53)	9 days (0 days to 2.7 years)
Age at second surgical palliation (n=48)	6.4 months (13 days to 11 years)
Age at third surgical palliation (n=21)	3.3 years (0.5 years to 15 years)
Mortality	14 (24.6)
Age at death	3.42 years (1 day to 23 years)

Median age at initial palliation was nine days, the second palliative surgery was at 6.4 months, and the third palliative surgery was at 3.3 years. The mean platelet count before initial surgical palliation was 349.21 ± 149.28 x 10^9^/L, with 15.8% of children having thrombocytosis. The mean platelet count on postoperative day 1 following the initial surgical palliation was 248.63 ± 126.48 x 10^9^/L. The mean platelet count prior to the second surgical palliation was 422.03 ± 135.54 x 10^9^/L, with 23.6% children having thrombocytosis. The mean platelet count on postoperative day 1 following the second surgical palliation was 217.13 ± 103.74 x 10^9^/L. The mean platelet count prior to the third surgical palliation was 351.36 ± 77.79 x 10^9^/L, with no children having thrombocytosis. The mean platelet count on postoperative day 1 after the third surgical palliation was 180.68 ± 73.83 x 10^9^/L. The mean platelet count at the most recent follow-up in patients at least one year removed from the third surgical palliation was 264.12 ± 113.39 x 10^9^/L, with none having thrombocytosis (Tables 2-4). There was no significant difference in platelet counts between those with right and left isomerism at any stage.

**Table 2:** Platelet counts Mean platelet count for at each time point †Significant difference between this value and value immediately preceding this ‡Significant difference between this value and the value before stage 1

Platelet count before first palliation	Platelet count after first palliation	Platelet count before second palliation	Platelet count after second palliation	Platelet count before third palliation	Platelet count after third palliation	Platelet count late after third palliation
349.21 ± 149.28	248.63 ± 126.48†	422.03 ± 135.54†	217.13 ± 103.74†	351.36 ± 77.79†	180.68 ± 73.83†	264.12 ± 113.39†

**Table 3:** Univariate analysis Univariate analysis to idnetify chracteristics associated with thrombocytosis prior to iniital surgical palliation

No Thrombocytosis Before Initial Palliation (n=48)	Thrombocytosis Before Initial Palliation (n=9)	Odds Ratio (95% Confidence Interval)	p-value
34 (70.4)	4 (40.0)	0.281 (0.039 to 2.014)	0.189
23 (48.1)	3 (60.0)	1.615 (0.232 to 11.263)	0.626
0 (0)	0 (0)	--	--
30 (63.0)	1 (20.0)	0.147 (0.014 to 1.506)	0.075
34 (70.4)	2 (40.0)	0.281 (0.039 to 2.014)	0.189
9 (18.5)	1 (20.0)	1.100 (0.100 to 12.087)	0.938
11 (22.2)	1 (20.0)	1.090 (0.084 to 11.457)	0.908
12 (25.9)	0 (0)	--	0.372
23 (48.1)	3 (60.0)	1.615 (0.232 to 11.263)	0.626
37 (77.8) 4 (7.4) 7 (14.8)	5 (100.0) 0 (0) 0 (0)	-- -- --	0.505
37 (77.8) 11 (22.2)	2 (40.0) 3 (60.0)	-- --	0.399

**Table 4:** Univariate analysis Univariate analysis to identify characteristics associated with thrombocytosis before second palliation

	No Thrombocytosis Before Second Palliation (n=29)	Thrombocytosis Before Second Palliation (n=9)	Odds Ratio (95% Confidence Interval)
Atrioventricular septal defect	19 (65.5)	5 (55.6)	0.658 (0.144 to 3.013)
Double outlet right ventricle	17 (58.6)	5 (55.6)	0.882 (0.195 to 3.987)
Double inlet left ventricle	0 (0)	0 (0)	--
Bilateral superior caval vein	15 (51.7)	4 (44.4)	0.747 (0.166 to 3.357)
Left superior caval vein	18 (62.1)	5 (55.6)	0.833 (0.164 to 3.425)
Interrupted inferior caval vein	6 (20.7)	1 (11.1)	0.479 (0.050 to 4.614)
Discordant atrioventricular connections	3 (10.3)	3 (33.3)	4.34 (0.780 to 6.948)
Discordant ventriculoarterial connections	7 (24.1)	1 (11.1)	0.393 (0.046 to 4.214)
Anomalous pulmonary venous connection	12 (43.3)	1 (11.1)	0.177 (0.024 to 0.956)
Splenic anatomy			
Absence of spleen	19 (65.5)	9 (100.0)	--
Multiple spleens	5 (17.2)	0 (0)	--
Solitary, normally located spleen	5 (17.2)	0 (0)	--
Atrial appendage morphology			
Right	20 (69.0)	7 (77.8)	--
Left	9 (31.0)	2 (22.2)	--

When comparing platelet counts at the various stages to those prior to initial surgical palliation, platelet counts at no subsequent time point differed significantly. When comparing platelet counts at the various stages to the platelet count immediately preceding it, significant differences were noted between all stages and the stage immediately preceding each one.

Table 5 outlines the platelet count at various stages in those with thrombocytosis prior to initial surgical palliation. Mean platelet count before the initial surgical palliation was 607.40 ± 105.26 x 10^9^/L with a decrease in mean platelet count to 288.33 ± 163.30 x 10^9^/L after the third surgical palliation.

**Table 5:** Platelet counts Mean platelet count at each time for those who had thrombocytosis prior to initial surgical palliation †Significant difference between this value and value immediately preceding this ‡Significant difference between this value and the value before Stage 1

Platelet count before initial palliation	Platelet count after initial palliation	Platelet count before second palliation	Platelet count after second palliation	Platelet count before third palliation	Platelet count after third palliation	Platelet count late after third palliation
607.40 ± 105.26	331.20 ± 125.14†	452.20 ± 122.63	193.00 ± 81.25†	355.67 ± 83.20†	196.33 ± 40.15	288.33 ± 163.30

Univariate analysis of those with and without thrombocytosis before initial surgical palliation did not identify any particular cardiac anomalies, splenic anatomy, or isomerism sidedness as being associated with increased risk of thrombocytosis. Multivariate analysis demonstrated similar findings. Univariate analysis of those with and without thrombocytosis before the second surgical palliation identified discordant atrioventricular connections as being associated with thrombocytosis, with an odds ratio of 4.34. This did not remain significant in the multivariate analysis.

## Discussion

Platelet counts are dynamic in children with functionally univentricular hearts and isomerism. This is not unexpected as these children undergo multiple procedures early in life, which in and of itself, can lead to changes in platelet count. What is unique to the isomerism population, however, is the increased risk of thrombocytosis. We demonstrated a 15.8% frequency of thrombocytosis before the initial surgical palliation and 23.6% before the second surgical palliation. No thrombocytosis was noted after the second surgical palliation.

No anatomic features could be identified as being predictive of thrombocytosis before the initial surgical palliation. Those with discordant atrioventricular connections tended to have an increased likelihood of thrombocytosis before the second surgical palliation, although this no longer remained significant after multivariate analysis.

In both the overall cohort, as well as only those with thrombocytosis before initial surgical palliation, there was an overall negative trend in the platelet count from prior to initial surgical palliation to after the third surgical palliation, although this was not statistically significant.

None of the anatomic characteristics studies were found to be predictive of thrombocytosis prior to initial surgical palliation. Prior to the second surgical palliation, however, discordant atrioventricular connections were an univariate predictor of thrombocytosis, although no multivariate predictors were identified.

Thrombocytosis in these children is of particular consequence as it has been demonstrated to increase the risk of aortopulmonary shunt thrombosis, a risk primarily noted with the Blalock-Taussig shunt. There is an increase in the risk of shunt thrombosis in children with functionally univentricular hearts who also have isomerism. This is particularly notable in those who have the absence of a spleen as well [12]. This risk has not been noted with the right ventricle to pulmonary artery conduits, and thus, the presence of thrombocytosis prior to initial surgical palliation should warrant increased consideration of the placement of a right ventricle to pulmonary artery shunt rather than a Blalock-Taussig shunt at the time of the initial surgical palliation.

Yamamura and colleagues demonstrated higher platelet counts in those with the absence of a spleen versus those with a spleen. The risk of thrombosis was also greater in those with an anatomic absence of the spleen [12]. It is important to note that those with isomerism and the presence of multiple spleens or a solitary, normally located spleen may also have functional asplenia, and thus, the anatomic splenic status may not be the ideal factor to use in such studies [11]. Splenic function based on Howell-Jolly bodies or pitted red blood cell counts would be the more appropriate factor to use. Our current study is limited by this as well as it had not previously been routine practice to assess splenic function. It would be prudent to routinely assess splenic function by means of either of the two aforementioned tests in all patients with isomerism for both evaluation of their infectious risk as well as to correlate with potential thrombocytosis.

The time course of thrombocytosis noted in those with isomerism is consistent with that found in those with functional asplenia due to other causes, such as sickle cell disease [16-17]. Splenectomy has been noted to be one of the main causes of reactive thrombocytosis. Post-splenectomy, platelet counts generally peak between postoperative day 7 and 20 and then return to normal within weeks or months, rarely taking years to normalize [16, 18].

It has now been demonstrated that at least 40% of those with isomerism will have ciliary dyskinesia [19]. In those with isomerism, the cilia often have normal ultrastructure but have an abnormal beating pattern and frequency, which compromises their function. Once thought to serve a mechanical function for moving fluids and other material, it is now understood that cilia also play a role in mechanosensory transduction [20]. Found in almost every tissue type, the role of cilia in several organs is still not well understood. Splenic tissue does contain cilia, and it is quite possible that functional asplenia present in the setting of a solitary spleen or multiple spleens may be the underlying mechanism of the abnormal function [20-22]. Whether this is due to alteration of a mechanical or sensory ciliary function is not clear.

This study is limited by its small sample size. Multivariate analysis is not highly powered due to this. Additionally, as there is variability in the underlying cardiac anatomy, not all patients underwent the same surgical palliation in the same order. Thus, those with hypoplastic left heart syndrome may have undergone a Norwood, Glenn, and Fontan operation while a child with double outlet right ventricle with pulmonary stenosis may have undergone Glenn and Fontan, not requiring an initial Norwood operation. This would explain why the ages of the initial, second, and third surgical palliations do not necessarily coincide with ages of the Norwood, Glenn, and Fontan that are generally seen. Nonetheless, this study does provide some insight into this phenomenon in a particularly vulnerable population while underscoring the need for larger studies, which would be best served by a multicenter approach.

## Conclusions

Thrombocytosis is not uncommon in the first year of life in those with isomerism and a functionally univentricular heart. Platelet counts decrease from birth onwards, however, and thrombocytosis at long-term follow-up after a third surgical palliation is uncommon. Independent risk factors for thrombocytosis could not be identified.
